# Effective Treatment of Postherpetic Neuralgia at the First Branch of the Trigeminal Nerve by High-Voltage Pulsed Radiofrequency

**DOI:** 10.3389/fneur.2021.746035

**Published:** 2021-10-11

**Authors:** Hongxi Li, Yuanyuan Ding, Yongqiang Zhu, Zhenkai Han, Peng Yao

**Affiliations:** Department of Pain Management, Shengjing Hospital of China Medical University, Shenyang, China

**Keywords:** postherpetic neuralgia, pulsed radiofrequency, pulsed radiofrequency parameters, ophthalmic branch of the trigeminal nerve, neuropathic pain

## Abstract

**Background:** Postherpetic neuralgia (PHN) is one of the most common and serious complications of herpes zoster. PHN of the first branch of the trigeminal nerve is painful and difficult to treat, as no definitive effective treatment is available. The aim of this retrospective study was to observe the efficacy and safety of treatment of PHN of the first branch of the trigeminal nerve with high-voltage pulsed radiofrequency (PRF) of the supraorbital nerve.

**Methods:** Fifty-two patients diagnosed with the PHN of the first branch of the trigeminal nerve at the Department of Pain Management, Shengjing Hospital, China Medical University, between April 2017 and October 2020 were selected. The PRF treatment of the supraorbital nerve was used. The patients were divided into two groups according to the treatment received: group C, conventional PRF group; and group H, high-voltage PRF group. The basic conditions, pain scores, and SF-36 scores of patients before treatment were recorded. Also, intraoperative and postoperative adverse events, visual analog scale (VAS) scores, 36-Item Short Form Health Survey (SF-36) scores at 1 week, 1 month, 3 months, and 6 months of follow-up were recorded. Furthermore, treatment efficiency was followed up at 6 months after treatment.

**Results:** The VAS scores of patients in both groups were significantly lower at all time points after treatment compared with presurgery. VAS scores in group H were lower than those in group C 1, 3, and 6 months after treatment. SF36 scores of patients in group H were better than those in group C 1, 3, and 6 months after treatment. The treatment efficiency at 6 months after treatment was higher in group H than in group C. No serious adverse events occurred in both groups.

**Conclusion:** The efficacy of the high-voltage PRF of the supraorbital nerve in treating the PHN of the first branch of the trigeminal nerve was superior to that of conventional PRF. It was a safe and effective treatment method.

## Introduction

PHN is a common, yet troublesome to treat, neuropathic pain (1, 2). In recent years, the incidence of PHN has increased yearly with the incidence of herpes zoster (3, 4). The incidence of PHN in the United States has been reported to be as high as 57.5 cases/(100,000 persons/year) (4). In China, the incidence of PHN is even higher, with statistics pointing to an incidence of 2.3% (5). The nature of pain in PHN is severe pinprick-like, burning, or electric shock-like pain in the lesion area, leading to unbearable and excruciating pain, which seriously affects the patient's quality of life and work. Current treatment options mainly include the use of analgesic drugs (6), nerve blocks (7), radiofrequency therapy (8), analgesic pump implantation (9), and spinal cord electrical stimulation (10). In elderly patients, especially those with multiple concomitant diseases, the side effects of medications limit the use of analgesic drugs (11). Patients with intractable pain even after conservative treatment require invasive treatments (12).

Conclusions regarding the effectiveness of PRF remain controversial. Evidence for the effectiveness and safety of PRF for neuropathic pain is mainly based on studies with small sample sizes and low quality (13). Some studies have also reported good pain relief with PRF in PHN (14). It is considered to be more effective compared with continuous radiofrequency (15). The main advantage of PRF is that it does not rely on the thermal destruction of nerve tissue but acts through electric fields that cause only transient mild edema without affecting the structural integrity of the nerve, which serves as a modulator of the nerve (16, 17). Conventional radiofrequency (CRF) produces neurodestructive effects, and CRF treatment increases the risk of thermal injury or nerve damage, further exacerbating neuropathic pain (18). Therefore, PRF is more suitable than CRF for the minimally invasive treatment of PHN. However, the effectiveness and safety of PRF treatment for the first branch of the trigeminal nerve PHN has not been clearly reported.

The results of our previous study showed that high-voltage PRF could treat PHN in the thoracolumbar region well, and the effect of 65 V high-voltage PRF in relieving PHN was significantly better than that of 45 V and 55 V (8). However, the effectiveness of treatment for the first branch of the trigeminal nerve PHN is unknown. The purpose of this study was to observe the effectiveness and safety of high-voltage vs. conventional PRF for treating V1 PHN, with the aim of providing some guidance for clinical work.

## Materials and Methods

### Patients

**Figure 1:**
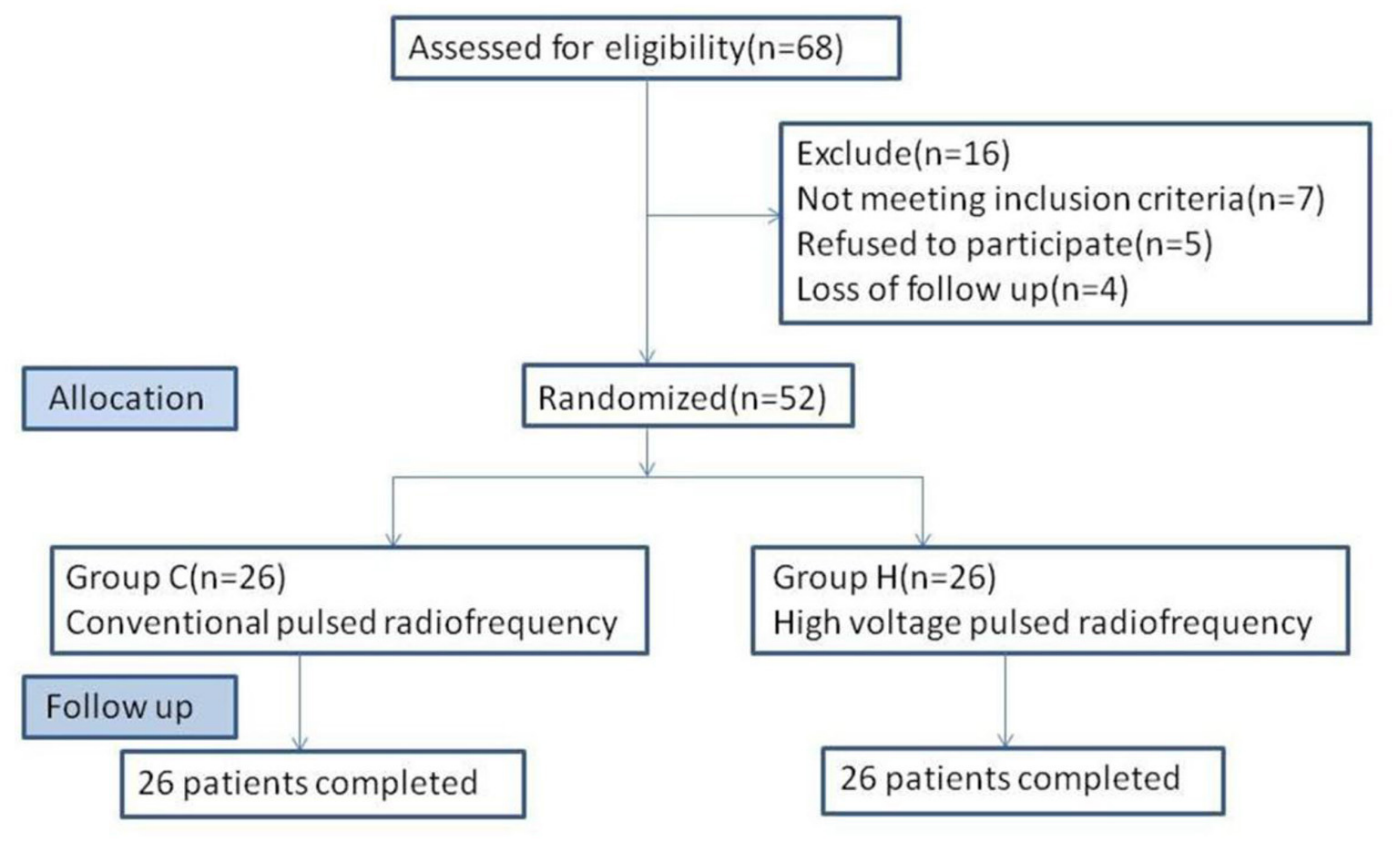
Flowchart of the study. Fifty-two patients were randomized to C group (*n* = 26) and H group (*n* = 26).

From January 2017 to October 2020, 52 patients with PHN at the first branch of trigeminal nerve, who met the inclusion criteria, were admitted to the pain department of Shengjing Hospital, China Medical University (Figure 1). All patients were given pharmacological and injectable treatments before treatment, which were not effective. Pharmacological treatment included pregabalin, analgesic drugs, and nerve-nourishing drugs, and the treatment protocol was the same in both groups. Injection treatment was performed by supraorbital nerve injection; 1 mL of an analgesic solution (1 mL of 2% lidocaine + vitamin B12 0.5 mg + compound betamethasone 5 mg) was administered. However, pain relief was maintained for <3 days after the injection. Patients further received PRF treatment. Patients were randomly divided into two groups according to treatment modality: group C, conventional pulsed radiofrequency group (*n* = 26); and group H, high-voltage PRF group (*n* = 26). All patients had unilateral PHN. After PRF treatment, both groups received drug injections. The study was approved by the ethics committee of Shengjing Hospital, China Medical University. All patients were informed of the risks and complications and signed an informed consent form before treatment.

### Inclusion Criteria

(1) The patient was diagnosed with the ocular branch PHN; (2) The natural course of the disease was between 1 and 3 months; (3) The conventional treatment was ineffective, with a VAS score >5.

### Exclusion Criteria

(1) The participants had allergy and abuse of related drugs; (2) Diabetes, sequelae of cerebral thrombosis, severe cardiopulmonary disease, or severe liver and kidney dysfunction and other serious systemic diseases; (3) Mental illnesses that did not cooperate; (4) Obvious abnormalities in biochemical tests such as coagulation function; (5) Pregnant women.

### Surgical Procedure

**Figure 2:**
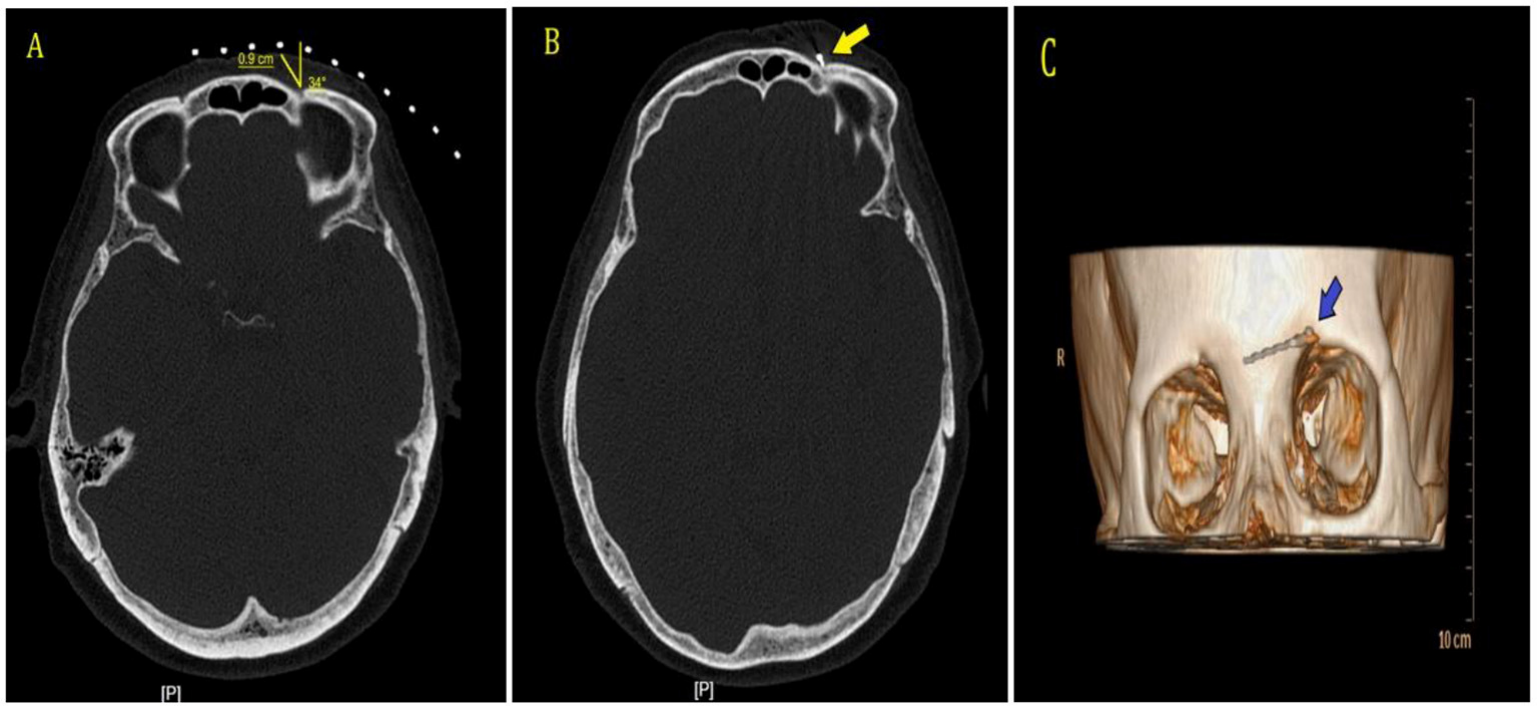
CT guidance and 3D reconstruction. **(A)** CT scan was performed to establish puncture point and needle path. **(B)** CT scan showed that the radiofrequency needle was located in the left supraorbital foramen, shown by the arrow. **(C)** 3D-CT showed the radiofrequency needle was located in the left supraorbital foramen, shown by the arrow.

The patients were placed supine on the computer tomography (CT) bed, and their heart rate, oxygen saturation, and blood pressure were routinely monitored. The electrode plates of the radiofrequency device were attached to their ipsilateral shoulders. Only the treatment site was exposed, while the rest of the body was covered with a lead safety suit for protection against radiation shielding. CT scanning was performed to locate the supraorbital foramen or supraorbital notch on the side of the lesion. The puncture path was developed and routinely disinfected, and a sterile sheet was placed. After local anesthesia with 0.5% lidocaine, the radiofrequency needle (21 G, length 100 mm and length of the active tip 5 mm) was gradually inserted at a predetermined angle and depth, and the position of the needle tip was adjusted under the guidance of 3D CT reconstruction. to confirm that the radiofrequency needle was located in the supraorbital foramen or supraorbital notch, and the patient appeared to have radiological sensation (Figure 2). The radiofrequency treatment instrument (COSMAN MEDICAL INC., Burlington, US) was connected and tuned to the sensory test mode (50 Hz, 1.0 ms). The position of the needle tip was adjusted with a stimulation current of 0.1–0.2 V to elicit the corresponding forehead and parietal painful heteroesthesia, covering the patient's pain area. It is necessary to avoid puncturing the needle too deeply into the supraorbital foramen. After confirming the position, the RF instrument was connected. For patients in group C, an automatic pulse mode was used with parameters set as follows: temperature, 42°C; frequency, 2 Hz; pulse width, 20 ms; and time, 900 s. For patients in group H, a manual pulse mode was applied with parameters as follows: initial voltage of 45 V, gradually increased to a maximum voltage of 65 V; temperature control below 50°C; and pulse RF time, 900 s. At the end of treatment, patients in both groups were given 1 mL of drug injection in the supraorbital nerve (1 mL of 2% lidocaine + vitamin B12 0.5 mg + compound betamethasone 5 mg). After removing the puncture needle and applying the sterile dressing, the patients were postoperatively bedridden for 6 h.

### Efficacy Evaluation and Follow-Up

The general conditions of the patients included gender, age, weight, pain duration, pain site, VAS score, and anticonvulsant dose. The follow-up periods were 1 week, 1 month, 3 months, and 6 months after operative. The “blinded” follow-up visits were performed by nontreatment-specific physicians without knowledge of the patient group using a telephone. The following parameters were assessed.

#### 1. VAS

VAS was used to assess pain. It was recorded before treatment and at the time of 1 week and 1, 3, 6 months after treatment.

#### 2. SF-36

The health questionnaire (SF-36) was used to assess the quality of life of patients (19, 20). The SF-36 included 36 items with eight dimensions: physical functioning, social functioning, physical role, bodily pain, mental health, role emotion, vitality, and general health. The values of physical component summary (PCS) and mental component summary (MCS) were calculated to evaluate quality of life. The higher the score, the higher the quality of life. It was recorded before treatment and at the time of 1 week and 1, 3, 6 months after treatment.

#### 3. Treatment Efficiency

Treatment efficiency was defined as ≥50% reduction in the VAS score at 6 months after treatment compared with presurgery.

#### 4. Adverse Events

Intraoperative and postoperative adverse events were recorded, including temporary events (infection, hematoma, abnormal heart rhythm, etc.) and permanent events (corneal injury, nerve injury, etc.).

### Statistical Analysis

Statistical analysis was performed using SPSS22.0 software (IBM Corporation, Armonk, NY). Quantitative data were presented as mean ± SD, and qualitative data were described using frequencies and percentages. Independent sample *t*-test was used for comparison between groups; One-Way analysis of variance (ANOVA) was used for intra group comparison Count data were analyzed with the Chi-square test; Rank sum test was used to compare ranked data. *P* < 0.05 was considered statistically significant.

## Results

### Basic Information of Patients Before Operative

The basic information of patients in both groups included gender, age, weight, pain duration, pain side, preoperative medication dosage, and number of patients with ptosis symptoms. No statistically significant differences in basic information were found between the two groups (*P* > 0.05) (Table 1).

**Table 1:** The general conditions of the patients (mean ± SD).

**Parameters**	**Group C**	**Group H**
Patients (n)	26	26
Gender (M/F)	10/16	12/14
Age (year, range)	64.15 ± 12.29	66.62 ± 8.21
Wight (kg)	65.17 ± 10.88	66.00 ± 14.42
Pain duration (day)	56.69 ± 13.70	58.85 ± 16.62
Pain side (n,%)		
Left	10	8
Right	16	18
Preoperative drug dosage		
Pregabalin(mg/day)	317.31 ± 88.25	334.62 ± 88.25
Ptosis	3	3

*Group C, conventional pulsed radiofrequency; Group H, high voltage pulsed radiofrequency*.

### VAS Scores Before and After Treatment

**Figure 3:**
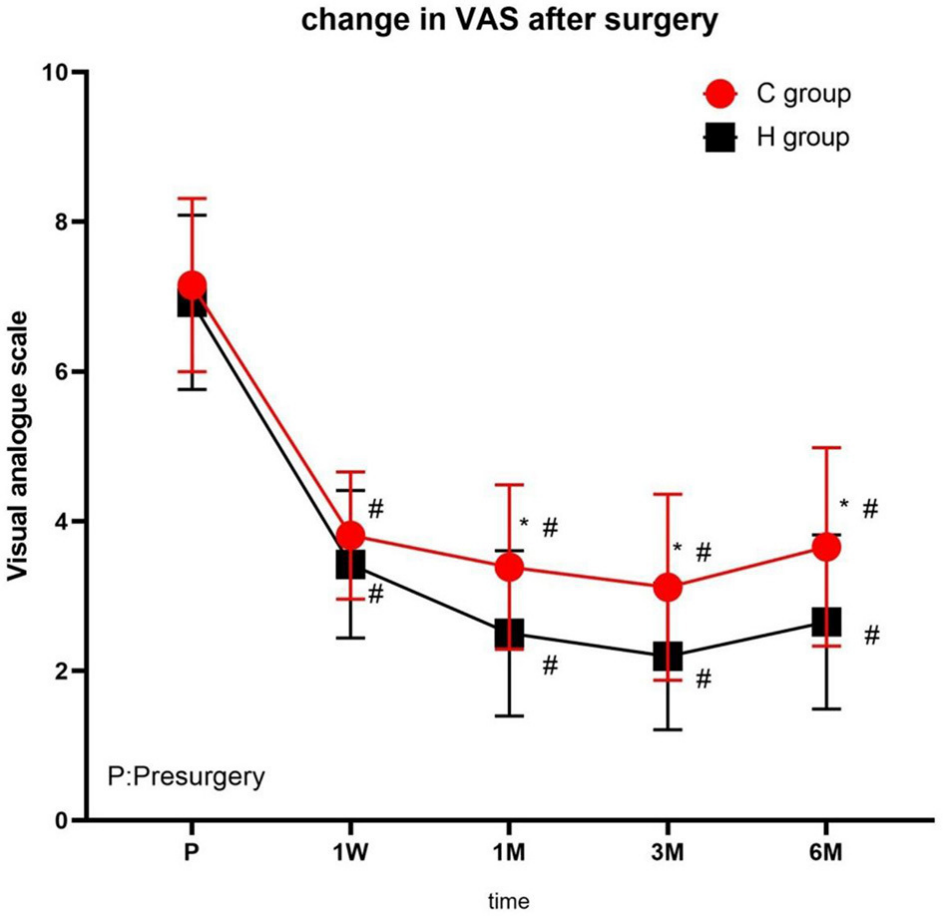
The comparison of VAS scores preoperative and postoperative in two groups. Results are represented as mean ± SD. #Compared to preoperative, *P* < 0.05; *Compared with C group, *P* < 0.05.

No significant difference was observed in VAS scores of the two groups before treatment. At each time point after treatment, the VAS scores were significantly different from the presurgery VAS scores of the two groups (1 week, 1, 3, and 6 months; *P* < 0.05), with the lowest scores at 3 months after treatment and a slight increase at 6 months. At 1, 3, and 6 months after treatment, the VAS scores in group H were significantly lower than those in group C (Figure 3 and Table 2).

**Table 2:** VAS scores before and after treatment.

**VAS scores**	**Group C**	**Group H**	***P* value**
Presurgery	7.15 ± 1.16	6.92 ± 1.16	0.476
1 week after treatment	3.81 ± 0.85	3.42 ± 0.99	0.138
1 month after treatment	3.38 ± 1.10^*^	2.50 ± 1.10	0.006
3 months after treatment	3.12 ± 1.24^*^	2.19 ± 0.98	0.005
6 months after treatment	3.65 ± 1.32^*^	2.65 ± 1.16	0.006

* *Compared with C group, P < 0.05*.

### SF-36 Scores Before and After Treatment

**Figure 4:**
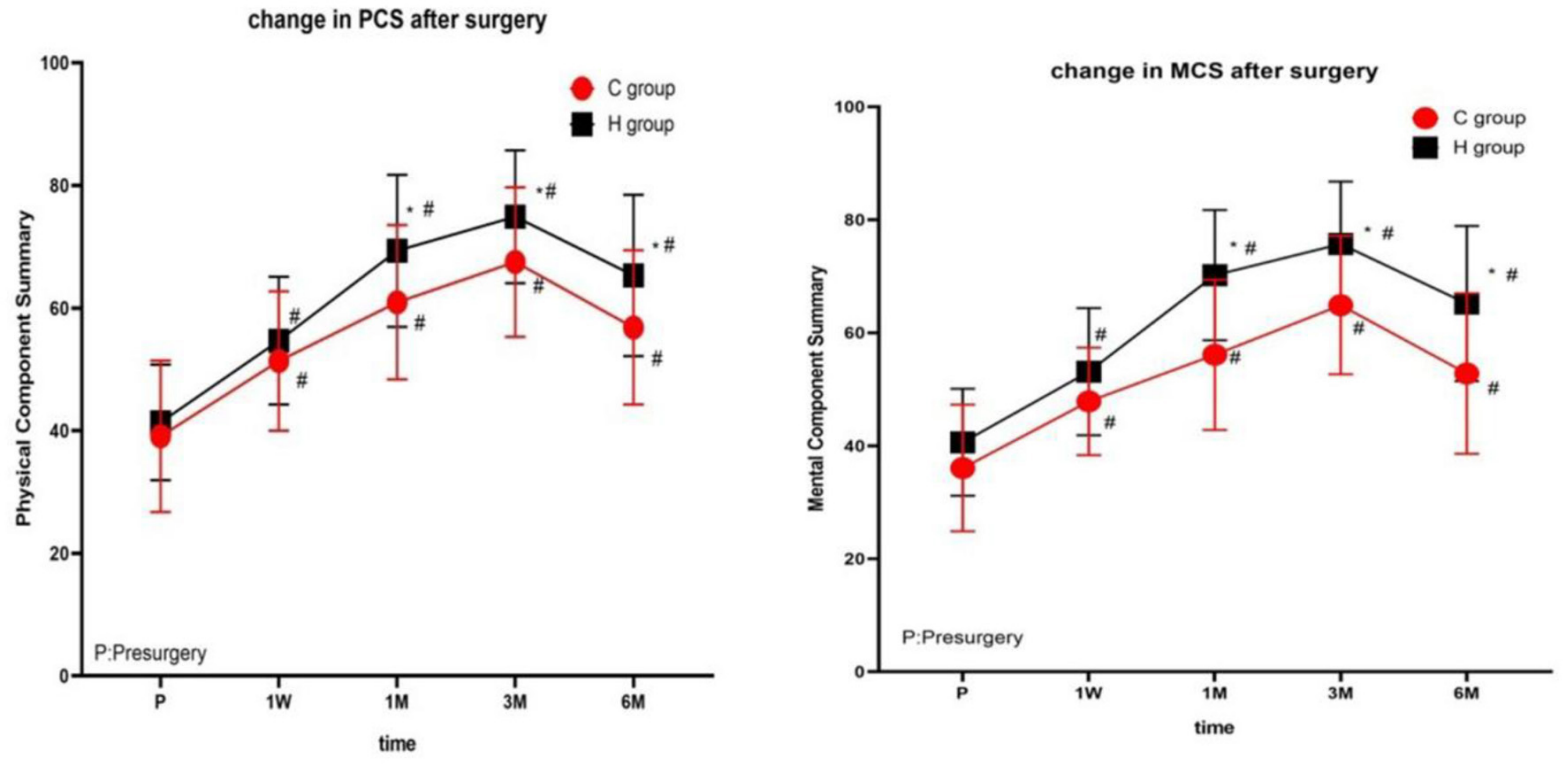
The comparison of quality of life scores (SF-36) preoperative and postoperative in two groups. PCS, physical component summary; MCS, mental component summary. Results are represented as means ± SD. #Compared to preoperative, P < 0.05; *Compared with group C, *P* < 0.05.

There were no significant differences in PCS and MCS scores between the two groups before treatment. At each time point after treatment, the PCA and MCS scores of the two groups were significantly different from those before treatment (1 week, 1, 3, and 6 months), reaching the highest at 3 months after treatment, and decreasing at 6 months after treatment (Figure 4 and Table 3).

**Table 3:** SF-36 scores before and after treatment.

**SF36**	**Group**	**Presurgery**	**1 week after treatment**	**1 month after treatment**	**3 months after treatment**	**6 months after treatment**
PCS scores	C	39.08 ± 12.35	51.35 ± 11.38	60.94 ± 12.57	67.51 ± 12.19	56.84 ± 12.60
	H	41.34 ± 9.44	54.67 ± 10.42	69.34 ± 12.40^*^	74.89 ± 10.83^*^	65.31 ± 13.14^*^
	P value	0.462	0.277	0.019	0.025	0.021
MCS scores	C	36.07 ± 11.21	47.86 ± 9.50	56.09 ± 13.29	64.89 ± 12.23	52.80 ± 14.21
	H	40.61 ± 9.46	53.11 ± 11.26	70.21 ± 11.53^*^	75.71 ± 11.04^*^	65.18 ± 13.73^*^
	P value	0.121	0.075	0.000	0.002	0.002

* *Compared with C group, P < 0.05*.

### Treatment Efficiency

At 6 months after treatment, the treatment efficiency rate in group H was 84.62%, and in group C was 53.84%. The treatment efficiency rate in group H was significantly higher than that in group C (*p* < 0.05) (Table 4).

**Table 4:** Treatment efficiency at 6 months after treatment (*n*, %).

**Parameters**	**Group C**	**Group H**	***P***
Patients (*n*)	26	26	
Treatment efficiency (*n*, %)	14(53.84%)	22(84.62%)^*^	0.034

* *Compared with C group, P < 0.05*.

### Adverse Events

Two cases of tachycardia occurred in group H and one case in group C, and 1 case of bradycardia occurred in both groups during treatment, these symptoms were relieved with prompt symptomatic treatments. One case of puncture site swelling occurred in both groups after treatment, which gradually subsided at 3 days. Two cases of pain aggravation occurred in group H at 1–2 days postoperatively and one case in group C, which gradually relieved within 3 days without medication. There were no permanent complications occurred in two groups. No statistically significant difference was found in the occurrence of adverse events between the two groups of patients (*p* > 0.05) (Table 5).

**Table 5:** Adverse events in Group C and Group H.

**Parameters**	**Group C**	**Group H**
Adverse reactions of local anesthetics (*n*)	0	0
Bradycardia (*n*)	1	1
Tachycardia (*n*)	1	2
Infection (*n*)	0	0
Swelling (*n*)	1	1
Worsened pain (*n*)	1	2
Ocular anesthesia	0	0
Corneal abrasions	0	0

No statistically significant difference was found in the occurrence of complications between the two groups of patients (Table 5).

## Discussion

In this study, both conventional PRF and high-voltage PRF are applied to relieve the pain of PHN and improve patients' quality of life to different extents. However, high-voltage pulsed therapy for ocular branch PHN showed lower VAS scores, higher SF36 scores at 1, 3, and 6 months after treatment. At 6 months after treatment, treatment efficiency was as high as 84.62%, and no serious adverse reactions occurred. The results showed that high-voltage PRF relieves ocular branch PHN more effectively than conventional voltage PRF.

The theory of PRF is that the tip of the electrode delivers a large current density. This current can be applied to target tissue by delivering the current in very brief pulses, and the high-frequency current in one cycle causes the target tissue to receive high voltage and generate heat. The relatively long pause between pulses allows any heat to be generated to dissipate and thereby prevent the development of any thermal lesion (21). Heavner et al. studied PRF test on fresh egg white that the results showed that when the needle tip temperature was higher than 60°C, egg white produced typical coagulation necrosis, suggesting that attention should be paid to control the temperature of the needle tip during PRF to minimize the thermal damage to the tissue (22). In this study, the maximum voltage was controlled at 65V and the temperature was controlled below 50°C to avoid nerve injury caused by further increase of voltage and temperature (8). In this study, the VAS score of group H was lower than that of group C at 1, 3, and 6 months after the treatment, indicating that high-voltage PRF treatment of ocular branch PHN is more effective than conventional PRF. Moreover, the analgesic effect of the high-voltage group was more durable. At 6 months after treatment, the treatment efficiency of the patients in the H group was 84.62%, which was higher than the 53.84% of the C group. This was consistent with the results of previous studies (23, 24). Previous studies have shown that the greater the voltage means the greater the electric field strength, which can improve the analgesic effect of PRF (17, 25). In this study, patients in group H received higher voltage treatment without damaging the nerve, and obtained better pain relief. This study confirmed that the analgesic effect of PRF is due to strong electric field effect rather than temperature effect (21).

The SF-36 is a practical and widely-tested instrument for measuring health status and medical outcomes (26). SF36 was suitable for assessing the quality of life of patients with neuropathic pain (27, 28) and was also often used to evaluate the quality of life of patients with PHN (24). The results of this study showed that patients in the H group had higher PCA, MCS scores at 1, 3, and 6 months after treatment than in the C group (*P* < 0.05). The quality of life for patients in the high-voltage PRF group has improved dramatically, which is related to the better analgesic effect as we believe.

It has been reported that trigeminal PHN is treated through foramen ovale trigemina semilunar ganglion (29). However, the foramen ovale is located at the base of the skull, the operation needs to be inserted into the skull, and the position of the first branch of the trigeminal nerve is deep, so it is difficult to accurately puncture the first branch, which may lead to complications such as cerebrospinal fluid leakage, weakened corneal reflex and intracranial hemorrhage (30, 31). Peripheral nerve PRF has certain advantages of simple operation and low puncture risk (32). The study of KooHyun Kim et al. showed that PRF used in the treatment of PHN has the best effect within 90 days (33). Therefore, in this study, patients with a course of <3 months were selected for supraorbital nerve PRF treatment. In this study, other treatment parameters and methods of the two groups were the same except that the PRF voltage settings were different. Nerve block has the effects of anti-inflammatory, eliminating edema, blocking the conduction pathway and vicious cycle of pain, and improving local blood circulation and promoting the recovery of damaged nerve endings (34). Surgical puncture and PRF may cause transient nerve edema and pain aggravation (35), and PRF treatment has a slower onset (2). Thus, the combined application of PRF and nerve block in this study will achieve better therapeutic effects (36, 37).

In this study, all the patients had no serious complications and adverse events. Three patients developed tachycardia during the operation. We believe that the causes of these adverse reactions are related to the patient's nervousness and pain caused by the puncture, but the symptom improved after relieving patient's emotions and symptomatic analgesia. Two patients had bradycardia during operation and two patients had transient swelling after operation, which was considered to be related to the puncture operation. The operator pressed the eyeball to avoid the puncture injury to the eye, causing oculocardiac reflex, and the condition relieved after stopping the compression. Three cases of pain aggravation occurred after the treatment, which gradually subsided within 3 days without any manifestation of injury aggravation. This was considered to be related to nerve edema caused by the treatment puncture rather than nerve injury caused by treatment (35). Two patients in the high voltage group had reduced ptosis after the treatment, and one patient in the conventional group had reduced ptosis after the treatment, with no statistically significant difference.

This study also has shortcomings: First, it was a single-center study with relatively small sample size, so a further randomized controlled study with larger sample size is needed for better results. Second, patients were followed up only for 6 months after the treatment so multicenter studies with a long-term follow-up should be performed to validate the findings.

## Conclusions

High-voltage PRF for treating of PHN of the transsphenoidal branch of the trigeminal nerve was more effective than conventional PRF in relieving pain, gaining higher treatment efficiency and patients' satisfaction. In addition, no serious intraoperative or postoperative complications occurred.

## Data Availability Statement

The original contributions presented in the study are included in the article/supplementary material, further inquiries can be directed to the corresponding author.

## Ethics Statement

The studies involving human participants were reviewed and approved by the Ethics Committee of Shengjing Hospital, China Medical University. The patients/participants provided their written informed consent to participate in this study.

## Author Contributions

HL, PY, and YD: conception and design of the study. HL, ZH, and YZ: acquisition of data. HL and ZH: data analysis. HL and PY: drafting the manuscript. All authors contributed to the article and approved the submitted version.

## Funding

This study was supported by 345 Talent Project.

## Conflict of Interest

The authors declare that the research was conducted in the absence of any commercial or financial relationships that could be construed as a potential conflict of interest.

## Publisher's Note

All claims expressed in this article are solely those of the authors and do not necessarily represent those of their affiliated organizations, or those of the publisher, the editors and the reviewers. Any product that may be evaluated in this article, or claim that may be made by its manufacturer, is not guaranteed or endorsed by the publisher.
